# Effects of SMC1A on immune microenvironment and cancer stem cells in colon adenocarcinoma

**DOI:** 10.1002/cam4.5891

**Published:** 2023-04-25

**Authors:** Jin Li, Qian Zhou, Li Liu, Jingdong He

**Affiliations:** ^1^ Department of Oncology The Affiliated Huaian No.1 People's Hospital of Nanjing Medical University Huaian China; ^2^ The Affiliated Huaian No.1 People's Hospital of Nanjing Medical University Huaian China

**Keywords:** cancer stem cells, colorectal cancer, immune checkpoint inhibitor, immune microenvironment, SMC1A

## Abstract

**Background:**

Our previous study suggested that SMC1 has significant functions in colorectal cancer (CRC). However, few reports have shown the effects of structural maintenance of chromosomes 1 (SMC1A) on the immune microenvironment and tumor stem cells.

**Methods:**

The Cancer Genome Atlas (TCGA) database, CPTAC database, Human Protein Atlas (HPA) database, the Cancer Cell Line Encyclopedia (CCLE) and Tumor Immune Single‐cell Hub were used. Flow cytometry and immunohistochemical analysis were checked for immune infiltration on MC38 mice model. Human CRC tissues were tested with RT‐qPCR.

**Results:**

The mRNA and protein levels of SMC1A were increased in colon adenocarcinoma (COAD) samples. SMC1A was associated with DNA activity. Interestingly, SMC1A was highly expressed in many types of immune cells at single‐cell levels. Moreover, the high expression of SMC1A was positively correlated with immune infiltration, and immunohistochemical analysis showed that SMC1A was positively associated with CD45 expression in MC38 mice model. Also, the percentage of IL4^+^CD4^+^ T cells (Th2) and FoxP3^+^CD4^+^ T cells (Tregs) was significantly higher in the SMC1A overexpression group than in control by flow cytometry assay in vivo. SMC1A expression could affect the proliferation of T cells in the mice model. The mutation and somatic cell copy number variation (SCNV) of SMC1A were also associated with immune cell infiltration. In addition to SMC1A in the “hot” T‐cell inflammatory microenvironment of colon cancer, SMC1A also positively correlates with the immune checkpoint genes CD274, CTLA4, and PDCD1 in colon adenocarcinoma (COAD) samples. Furthermore, we also found that SMC1A plays a positive correlation with the induction of cancer stem cells (CSCs). Our results also showed that miR‐23b‐3p binds SMC1A.

**Conclusion:**

SMC1A may be a bidirectional target switch that simultaneously regulates the immune microenvironment and tumor stem cells. Moreover, SMC1A may be a biomarker for the prediction of immune checkpoint inhibitor (ICI) therapy.

## Graphical abstract

In this study, SMC1A has the potential to serve as a bidirectional target switch, regulating both the immune microenvironment and cancer stem cells simultaneously. Additionally, SMC1A may function as a biomarker for predicting the effectiveness of immune checkpoint inhibitor (ICI) therapy.

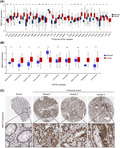

## 1 INTRODUCTION

CRC is one of the most common gastrointestinal malignancies.^1^, ^2^ About 15% of patients with CRC are initially diagnosed with stage IV, and about 40%–50% of patients rapidly suffer from recurrence and metastasis despite radical surgery. For CRC patients with advanced stages and recurrent metastases, the clinical outcome is poor and the overall survival time of patients is very short.^3^ Therefore, an in‐depth study of the mechanism of the occurrence and development of CRC will provide theoretical basic research for prognosis determination, early diagnosis and treatment, and the development of new therapeutic targets and treatment methods for CRC.

Chromosomal instability (CIN) is an important molecular type of colorectal carcinogenesis and development, accounting for 70%–85% of CRC. It has been found that chromosomal instability is also closely related to the aggressive, metastasis, and drug resistance of CRC.^4^, ^5^, ^6^ SMC1 is one of the subunits of endoglin (Cohesin complex), which interacts with chromatin at the end of mitosis and regulates the polymerization and separation of sister chromatids after DNA replication, and is important for maintaining chromosome stability.^7^ It has been shown that SMC1 is involved in several important aspects such as transcriptional regulation, DNA replication, and repair.^8^, ^9^ The aberrant expression of SMC1A serves critical roles in the development of multiple tumors. In glioma, knocking down the SMC1 gene resulted in blocking tumor cells in S phase and inhibiting the growth of tumor cells.^10^ SMC1 has the ability to promote metastasis of hepatocellular carcinoma and triple‐negative breast cancer, and overexpression of the SMC1 gene is associated with tumor progression and poor prognosis.^11^, ^12^, ^13^ In addition, mutations and aberrant expression of SMC1 gene are involved in the development of CRC.^14^

Our previous study also showed that SMC1 expression was significantly higher in colorectal cancer tissues than in normal or paraneoplastic tissues, and patients with high SMC1 expression had late stages, short survival, and poor prognosis.^15^ Furthermore, it was suggested that reducing SMC1 expression could reduce proliferation, increase apoptosis, and enhance sensitivity to oxaliplatin in CRC cells, suggesting that SMC1 may become a new target for the prognosis and treatment of CRC.^15^, ^16^ Another report also revealed that SMCA1 overexpression may be an adverse factor for prognosis in advanced CRC, and SMC1A knockdown inhibited the cell proliferation and cell cycle processes of CRC by blocking the activation of AKT and MAP kinases.^17^ Thus, we suggest that SMC1 has significant functions in CRC. However, few reports have shown the effects of SMC1A on the immune microenvironment and tumor stem cells.

## 2 MATERIALS AND METHODS

### 2.1 The mRNA expression of SMC1A

RNAseq data were downloaded from UCSC XENA (https://xenabrowser.net/datapages/) in transcripts per million reads (TPM) format for TCGA and The Genotype‐Tissue Expression (GTEx) and unified via the Toil process.^18^ The RNAseq data in TPM format were then log2 transformed. The ggplot2 package was used to visualize the expression of SMC1A in cancer and normal tissues, and the Mann–Whitney U test was used for statistical analysis. The pan‐cancer data of TCGA include 33 cancer types, and they are ACC (Adrenocortical carcinoma), BLCA (Bladder urothelial carcinoma), BRCA (Breast invasive carcinoma), CESC (Cervical squamous cell carcinoma), CHOL (Cholangiocarcinima), COAD (Colon adenocarcinoma), DLBC (Lymphoid Neoplasm Diffuse Large B‐cell Lymphoma), ESCA (Esophageal carcinoma), GBM (Glioblastoma multiforme), HNSC (Head and Neck squamous cell carcinoma), KICH (Kidney Chromophobe), KIRC (Kidney renal clear cell carcinoma), KIRP (Kidney renal papillary cell carcinoma), LAML (Acute myeloid leukemia), LGG (Brain lower grade glioma), LIHC (Liver hepatocellular carcinoma), LUAD (Lung adenocarcinoma), LUSC (Lung squamous cell carcinoma), MESO (Mesothelioma), OV (Ovarian serous cystadenocarcinoma), PAAD (Pancreatic adenocarcinoma), PCPG (Pheochromocytoma and Paraganglioma), PRAD (Prostate adenocarcinoma), READ (Rectum adenocarcinoma), SARC (Sarcoma), SKCM (Skin Cutaneous Melanoma), STAD (Stomach adenocarcinoma), TGCT (Testicular Germ Cell Tumors), THCA (Thyroid carcinoma), THYM (Thymoma), UCEC (Uterine Corpus Endometrial Carcinoma), UCS (Uterine Carcinosarcoma), and UVM (Uveal Melanoma).

### 2.2 Validation of protein expression of SMC1A

Protein levels of SMC1A in normal and CRC tumor tissues were verified by immunohistochemical (IHC) assays using the Human Protein Atlas database (HPA, https://www.proteinatlas.org/), which is a protein expression profiling IHC‐based database.^19^, ^20^, ^21^

### 2.3 Validation of the Cancer Cell Line Encyclopedia (CCLE) database

The CCLE database (https://sites.broadinstitute.org/ccle/) provides data on the genomes of over 1100 cell lines from different tumors, including a total of 56 intestinal cancer cell lines such as SW480, SW620, HCT116, etc.^22^ The CCLE database data are mainly obtained by high‐throughput sequencing, which contains five main dataset types, they are copy number, mRNA expression (Affymetrix), inverse phase protein array, reduced representation bisulfite sequencing, and mRNA expression (RNA sequencing). Using CCLE database, the genes associated with SMC1A were analyzed by Pearson and the final genes were filtered by Pearson correlation coefficient *r* > 0.5 and *p* value <0.001.

### 2.4 Gene Ontology (GO) and Kyoto Encyclopedia of Genes and Genomes (KEGG) analysis

Relevant genes obtained from the CCLE database were used for subsequent GO and KEEG pathway analysis, the org.Hs.eg.db R package was utilized for ID conversion, and the clusterProfiler R package was applied for GO and KEEG enrichment analysis.^23^ *p* values were adjusted using the BH method.

The differentially expressed genes in the high‐ and low‐risk groups of SMC1A of COAD were analyzed from the TCGA database. GO and KEGG enrichment analysis was performed using the clusterProfiler R package to obtain significantly enriched functions and pathways.^23^

### 2.5 SMC1A analysis at single‐cells levels

The Tumor Immune Single Cell Center (TISCH) database (http://tisch.comp‐genomics.org/) is a database focused on single‐cell RNA (scRNA) sequencing in the tumor microenvironment (TME).^24^ This database is used to study the expression levels of SMC1A in different immune cell types under single‐cell conditions in CRC. It was also analyzed for the expression of SMC1A in single cells in different TNM stages.

### 2.6 Immune cell infiltration analysis

Immune cell infiltration in COAD was detected by GSVA R package for the expression levels of SMC1A.^25^ The ssGSEA method was used to calculate immune cell infiltration. Spearman's method was used for correlation analysis, and a *p* value <0.05 was considered statistically significant.

### 2.7 Correlation of mutation and somatic cell copy number variation (SCNV) of SMC1A and immune cell infiltration

Tumor Immune Estimation Resource (TIMER) (https://cistrome.shinyapps.io/timer/) is a database for the systematic analysis of immune infiltration in different types of cancer.^26^, ^27^ The database is estimated by multiple methods of immune infiltration abundance for a comprehensive exploration of tumor immunological, clinical, and genomic features. The correlation of SMC1A mutation and SCNA status with immune cell infiltration in COAD was examined using the TIMER database. The two‐sided Wilcoxon rank‐sum test was used for statistical analysis.

### 2.8 Association of SMC1A expression and tumor stem cell scores

Tumor stem cell features were extracted from the transcriptome of COAD samples from TCGA.^28^ The correlation between SMC1A expression levels and tumor stemness scores was statistically analyzed by the Spearman test.

### 2.9 Mice and MC38 tumor model

A total of 10 female C57BL/6 mice, aged 6 weeks, were purchased from Beijing Vital River Laboratory Animal Technology Co., Ltd. The murine colon cancer cell line MC38 was maintained in DMEM (Gibco) supplemented with 10% fetal bovine serum (FBS, Gibco) and 1% penicillin/streptomycin, and cultured at 37°C with 5% CO2. Then, the control and SMC1A overexpressing MC38 cell lines were established according to our previous publication.^15^ Mice were inoculated subcutaneously in the flank with 5 × 10^5^ control or MC38 overexpressing cells. The mice were euthanized and tumors harvested when the maximum tumor diameter reached around 1.0 cm. All animal experiments were performed in accordance with the guidelines of the Institutional Animal Care and Use Committee of The Affiliated Huaian No.1 People's Hospital of Nanjing Medical University (IACUC‐1810008).

### 2.10 Immunohistochemical assay

Tumor tissues were fixed in 4% paraformaldehyde and embedded in paraffin. The slides (4 μm) were deparaffinized in xylene and hydrated using a graded alcohol series. Antigen retrieval was conducted in antigen unmasking solution at 100°C for 20 min. The sections were then incubated with 0.3% H_2_O_2_ for 20 min and blocked with 5% BSA (Sigma‐Aldrich) for 1 h at room temperature. The slides were incubated with primary antibodies (anti‐CD45, 1:50 dilution, BD Biosciences, 550,539) overnight at 4°C, followed by incubating with HRP‐conjugated IHC detection reagent for 2 h at 37°C. Immunoreactive cells were visualized using DAB, and nuclei were stained with hematoxylin for 15 s at room temperature. Cells were counted using an IX70 inverted fluorescence microscope.

### 2.11 Flow cytometry assay

Tumor samples are cut into small pieces and macerated through 70 μm cell strainer, then digested into single‐cell suspension by using digestive solution (1.5 mg/mL collagenase, 1.5 mg/mL hyaluronidase and 20 μg/mL DNase diluted by HBSS) at 37°C for 30 min. Cell suspensions were collected and lysed with RBC lysis buffer, then pretreated with Fc‐block CD16/CD32 antibody (BD Biosciences, 553,142) at 4°C for 15 min. For extracellular staining, cells were resuspended by FACS buffer (1% FBS 0.05% sodium azide in PBS), incubating with CD3 (FITC Hamster anti‐mouse, BD Biosciences, 561,827; 1:100 dilution) and CD4 (APC‐H7 Rat anti‐mouse, BD Biosciences, 560,246; 1:100 dilution) antibodies at 4°C for 20 min. For intracellular staining, cells were fixed in Fixation/Permeabilization Solution (eBioscience) for 15 min and stained with FoxP3 (PerCP‐Cy5.5 Rat anti‐Mouse, BD Biosciences, 563,902; 1:100 dilution) and Ki67 (PE Rat anti‐mouse, Biolegend, 16A8; 1:200 dilution) antibodies at 4°C for 20 min after washing. For cytokine expression, cells were resuspended using DMEM medium containing 10% FBS and 1% penicillin/streptomycin and activated with Leukocyte Activation Cocktail (BD Biosciences, 554,656; 1:100 dilution) at 4°C for 6 h, then stained with IL4 antibody (BV421 Rat anti‐mouse, Biosciences, 562,915). Cells were analyzed using a flow cytometer (BD Biosciences), and the data were analyzed using FlowJo software.

### 2.12 Patients and human CRC tissues

The samples of 37 CRC patients were collected from January 2019 to May 2021 at the Affiliated Huaian No.1 People's Hospital of Nanjing Medical University. Patients' tumors and adjacent normal paracancerous tissues were included in this study. The CRC diagnosis was confirmed by at least two pathologists. Collected CRC sample tissues (tumor tissues and paraneoplastic tissues) were preserved in RNA Stabilizing Tissue Preservation Solution (Beyotime Biotechnology, R0118) and stored at −80°C. All samples used in this project were de‐identified and assigned a study number. None of these 37 patients had received any preoperative anticancer therapies. All procedures involving human material in this study were approved by the committee of The Affiliated Huaian No.1 People's Hospital of Nanjing Medical University (Ethical number: YX‐2021‐106‐01).

### 2.13 RNA isolation and real‐time quantitative PCR (RT‐qPCR)

Total RNA of colon cancer samples was extracted with TRIzol (Invitrogen). To check the expression of SMC1A, 500 ng RNA was reverse transcribed to cDNA (Takara Biotechnology Co., Ltd) and then qPCR assays were performed. The primer sequences were as follows: SMC1A Forward: 5’‐GGAGCAGCAGCAGATTGAG‐3′, Reverse: 5’‐TCTCTTCTTCCATCCGTTCTTC‐3′; GAPDH Forward: 5’‐TGACTTCAACAGCGACACCCA‐3′, Reverse: 5’‐ACCCTGTTGCTGTAGCCAAA‐3′. The expression of SMC1A was normalized by GAPDH. To check the expression of has‐miR‐23b‐3p, 500 ng RNA was reverse transcribed to cDNA (QIAGEN; USA), then qPCR assays were performed using miRCURY LNA SYBR Green PCR kit (QIAGEN). The hsa‐miR‐23b‐3p (QIAGEN) and the normalization with U6 (QIAGEN) were used for primers of qPCR. The expression of has‐miR‐23b‐3p was normalized by U6. The fold changes of SMC1A and hsa‐miR‐23b‐3p were calculated using 2−^ΔΔCt^ method.

### 2.14 Statistical analysis

All statistical analyses were performed using R language 4.0.5 or SPSS 16.0 software. The expression of SMC1A in cancer and normal tissues of pan‐cancer was analyzed by the Wilcoxon rank‐sum test. The genes associated with SMC1A in CCLE database were analyzed by the Pearson's test. *p* values of GO and KEGG analysis were adjusted using the BH method. The expression of SMC1A in different TNM stages at single‐cell levels from TISCH database was analyzed by the Kruskal–Wallis test. The immune cell infiltration analysis was performed by the Spearman's test. The correlation of SMC1A mutation and SCNA status with immune cell infiltration in COAD was examined by the two‐sided Wilcoxon rank‐sum test. The correlation between SMC1A expression and the expression of immune checkpoint genes (CD274, CTLA4, and PDCD1) or the expression of stem cell indicators (CD133, CD29, CD166, CD44, Lgr5, and Oct4) was analyzed by the Spearman's test. Two‐tailed *t*‐tests were used to compare data between two groups in qRT‐PCR, Immunohistochemistry, and flow cytometry assay. *p* < 0.05 was considered to be statistically significant.

## 3 RESULTS

### 3.1 SMC1A is highly expressed in colon adenocarcinoma

**FIGURE 1:**
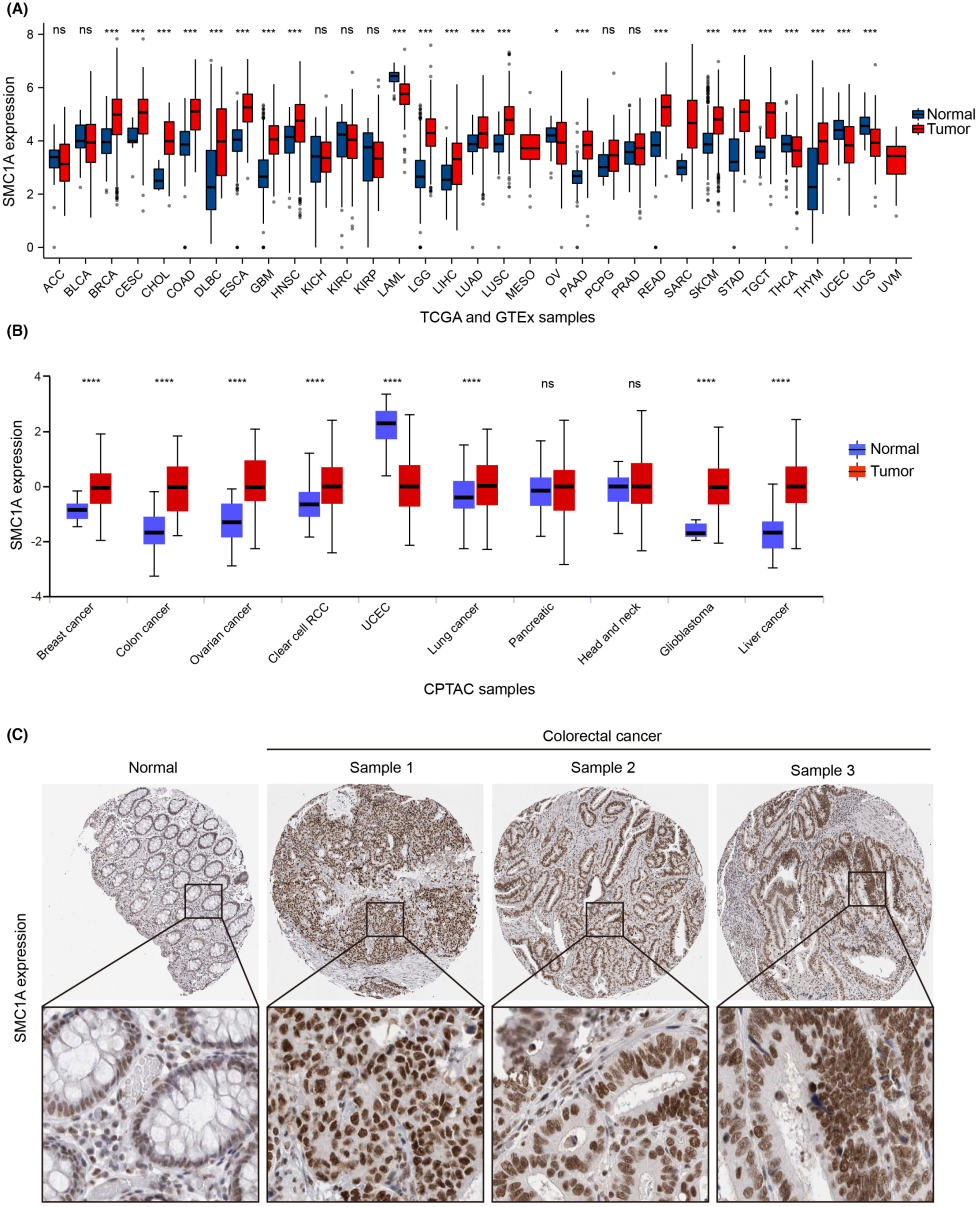
SMC1A is highly expressed in colon adenocarcinoma. (A) The mRNA expression of SMC1A was detected in all tumor samples and normal tissues of pan‐cancer from the TCGA and GTEx databases. (B) The SMC1A protein expression of colon cancer, breast cancer, ovarian cancer, clear cell renal cell carcinoma, and uterine corpus endometrial carcinoma (UCEC) from CPTAC Confirmatory/Discovery dataset was generated by UALCAN (http://ualcan.path.uab.edu/index.html). (C) Protein levels of SMC1A in normal and CRC tumor tissues were verified by immunohistochemical (IHC) assays by HPA database. SMC1A is mainly expressed in the nucleus. *p < 0.05; ***p < 0.001; ****p < 0.0001; ns—no significance.

To examine the involvement of SMC1A in clinical cancer patients, we first investigated the mRNA levels of SMC1A in all tumor samples and normal tissues in the TCGA and GTEx databases. Our findings demonstrated that SMC1A is an oncogene that is highly expressed in numerous clinical cancer tissues compared with normal tissues, including COAD (Figure 1A). Similarly, SMC1A protein expression was increased in colon cancer tissues in the mass spectrometry‐based proteomics of the CPTAC database (Figure 1B). To further identify the protein levels of SMC1A, we next examined the protein expression of SMC1A by IHC using the Human Protein Atlas database (HPA, https://www.proteinatlas.org/). SMC1A was mainly expressed in the nucleus (Figure 1B). Similar to the results of TCGA and CPTAC databases, the protein expression levels of SMC1A were significantly higher in tumor tissues of CRC than in normal tissues (Figure 1C). These results further confirmed that SMC1A may be a potential biomarker for COAD.

### 3.2 The functions and pathways associated with SMC1A

**FIGURE 2:**
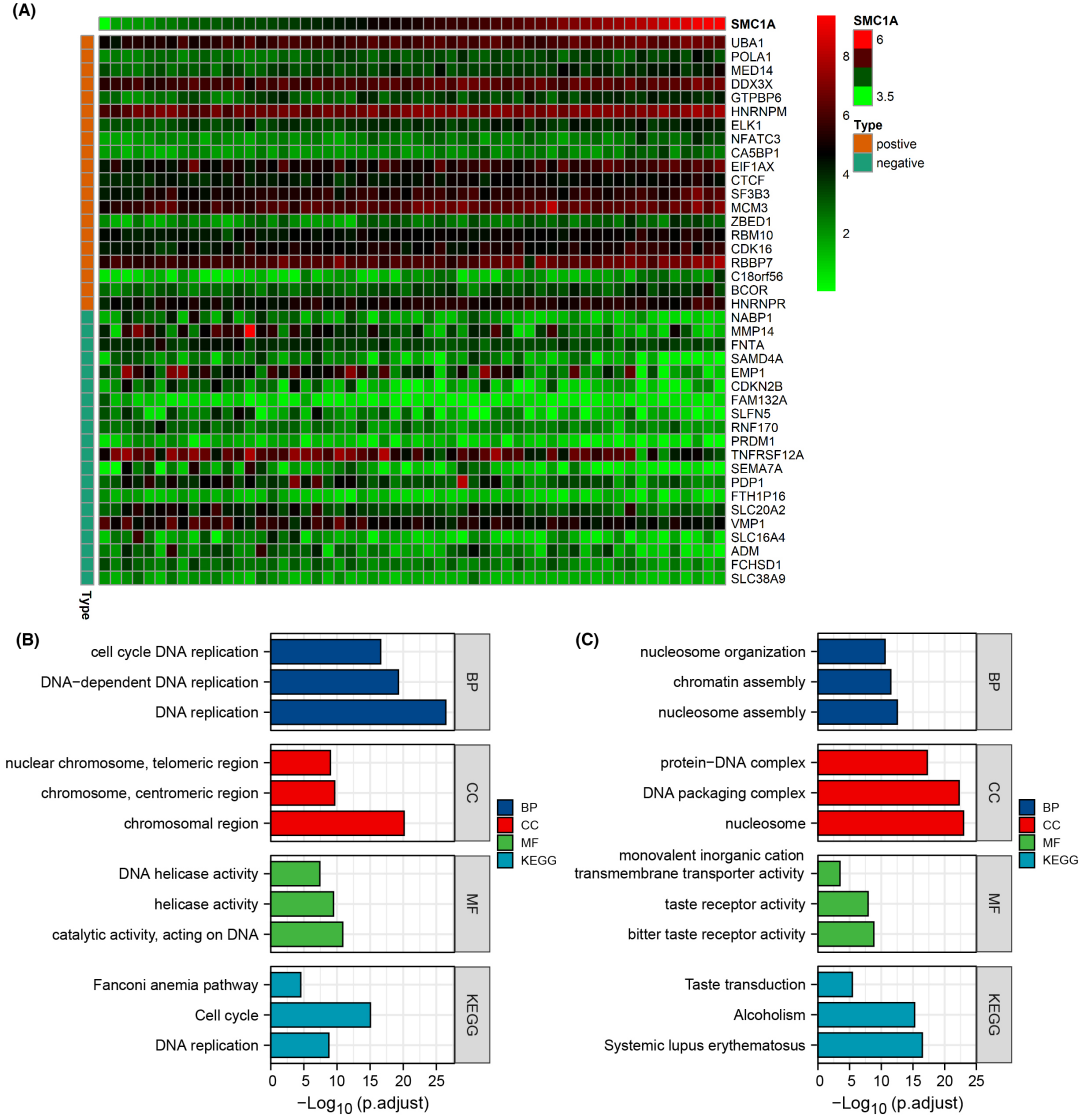
The functions and pathways associated with SMC1A. (A) Heat map showed the top 20 genes positively and negatively associated with SMC1A in the CCLE database. (B) GO and KEEG analyses were used to inspect the relevant functions and signaling pathways of SMC1A in the sequencing results of CRC cell lines from CCLE database. (C) GO and KEGG analyses were showed that SMC1A was associated with DNA activity from TCGA database.

Next, we researched the relevant functions and signaling pathways of SMC1A using gene ontology (GO) and KEEG pathways. First, we used the Cancer Cell Line Encyclopedia (CCLE) database (https://sites.broadinstitute.org/ccle/), which has sequencing results for 56 large intestine cell lines. We found SMC1A‐associated genes in 56 cell lines (by correlation coefficient >0.5, *p* value <0.001), and we showed the most positive and negative correlations with SMC1A in 20 genes, respectively (Figure 2A). GO and KEEG analysis showed that the most related to the expression of SMC1A was DNA activity, including DNA replication, DNA helicase activity, cell cycle, and DNA replication (Figure 2B). Moreover, we examined the differential genes of SMC1A in the TCGA database and analyzed the GO and KEGG pathways, which similarly showed that SMC1A was associated with DNA activity (Figure 2C).

### 3.3 SMC1A is expressed in a variety of immune cells

T cells are one of the most important components of the immune system and assume an essential function in the immune response to tumors. Recent studies have shown that chromatin structure‐mediated proliferation of T cells is one of the key mechanisms of immune regulation, specifically, resting T cells possess condensed chromatin, while proliferating T cells possess a more open chromatin structure.^29^ Chromosome structure maintenance complexes (SMCs) were found to play roles in the regulation of chromatin structure during cell division; however, they are now found to function in T‐cell development and function as well.^29^

**FIGURE 3:**
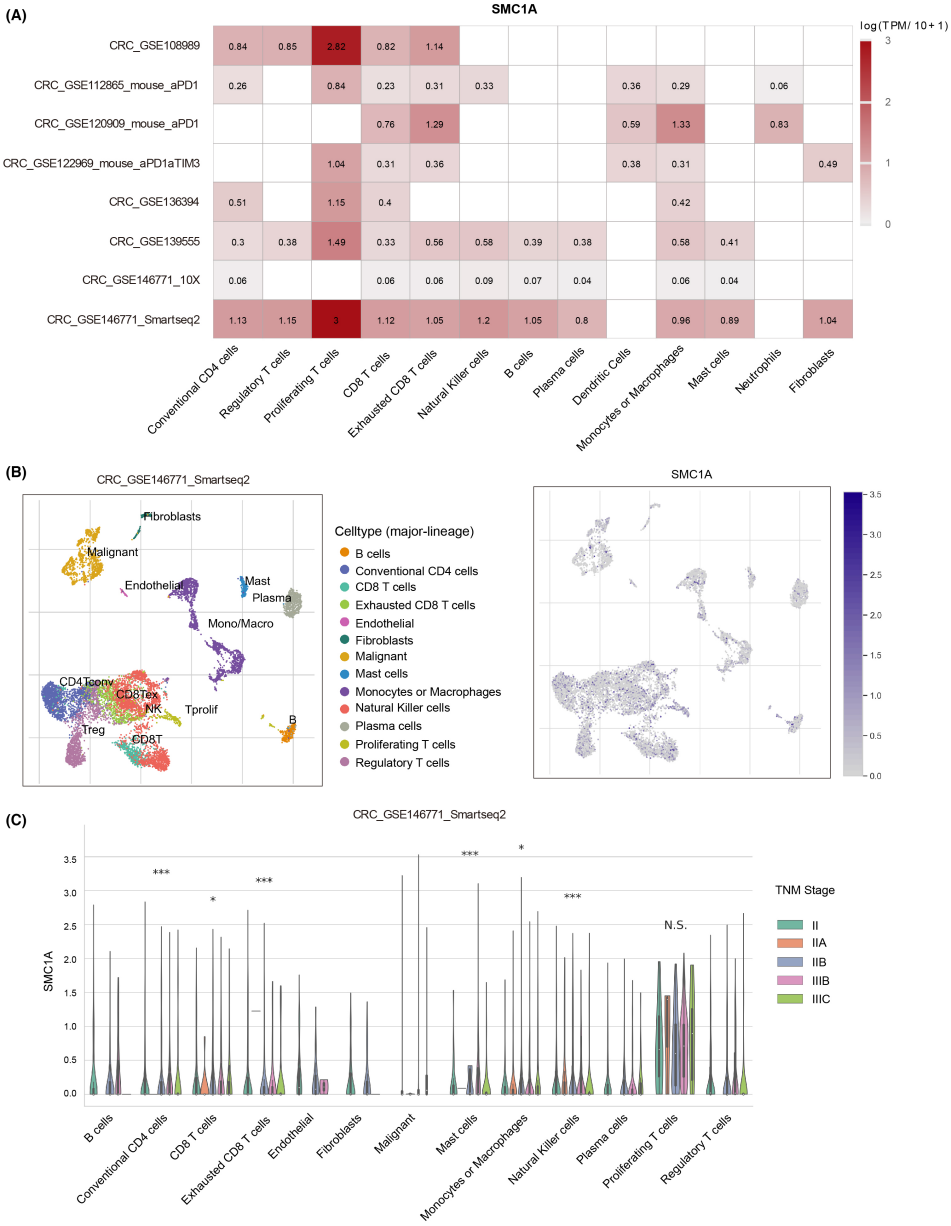
SMC1A is expressed in a variety of immune cells. (A) The expression of SMC1A at the single‐cell levels in different cell types of CRC samples using the TISCH database. (B) The expression levels of SMC1A in different immune cell types in a GSE database CRC_GSE146771_Smartseq2. (C) The SMC1A levels of multiple types of immune cells were associated with TNM staging, including conventional CD4 cells, CD8 T cells, exhausted CD8 T cells, mast cells, monocytes or macrophages, and natural killer cells. *p < 0.05; ***p < 0.001; ns—no significance.

Therefore, we next explored whether SMC1A serves significant functions in the immune microenvironment. To examine how SMC1A modulates the immune microenvironment in COAD, we first investigated the expression of SMC1A at the single‐cell levels in different cell types using the TISCH (Tumor Immune Single‐cell Hub) (http://tisch.comp‐genomics.org/home/; http://tisch.comp‐genomics.org/home/) database, and we found that SMC1A was highly expressed in many types of immune cells among several datasets (Figure 3A). Further, we explored the expression levels of SMC1A in different immune cell types in a GSE database CRC_GSE146771_Smartseq2, and the results showed that SMC1A was significantly expressed in a variety of immune cells (Figure 3B). In particular, among all types of immune cell types, SMC1A expression showed extremely high in proliferating T cells (Figure 3A,B). Interestingly, the SMC1A levels of multiple types of immune cells were associated with TNM staging, including conventional CD4 cells, CD8 T cells, exhausted CD8 T cells, mast cells, monocytes or macrophages, and natural killer cells (Figure 3C). However, the expression levels of SMC1A in proliferating T cells were not associated with TNM staging (Figure 3C).

### 3.4 Correlation between SMC1A and immune cell infiltration

**FIGURE 4:**
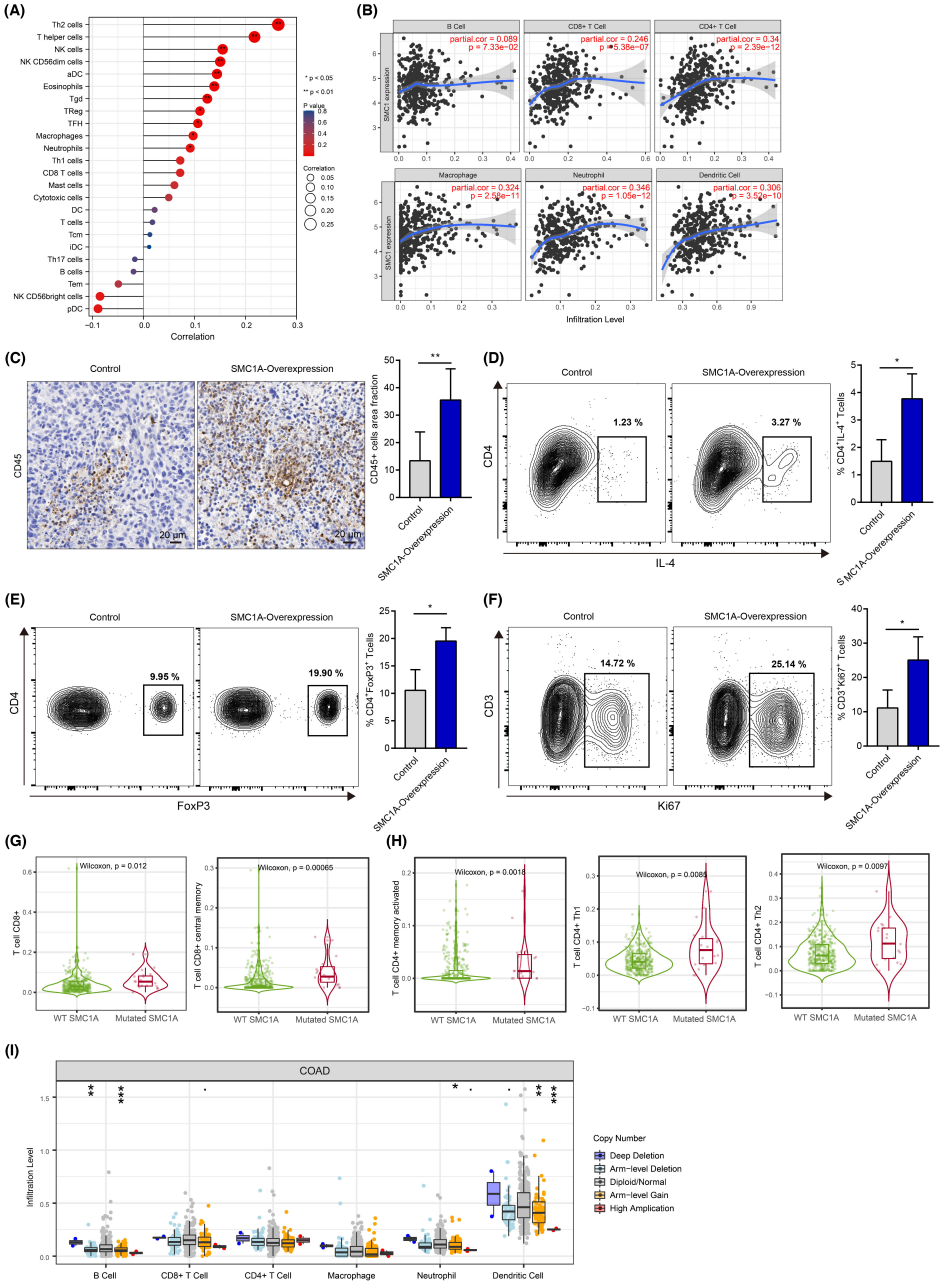
Correlation between SMC1A and immune cell infiltration. (A) The ssGSEA method calculated the correlation coefficient between SMC1A expression and immune cells from TCGA database. (B) High expression of SMC1A was positively correlated with immune infiltration. (C) Tumor tissues from the mice model were stained with CD45 antibody by immunohistochemistry assay. (D–F) Flow cytometry analysis showed the expression of IL4^+^CD4^+^ T cells, FoxP3^+^CD4^+^ T cells, and Ki67^+^CD3^+^ T cells in tumor samples from mice model. (G) The mutated SMC1A was elevated in CD8 T cells and CD8 central memory T cells. (H) CD4 T cells, CD4 T‐cell Th1, and Th2 expression were higher in the mutated SMC1A group than in the SMC1A wild‐type group. (I) The correlation between immune cell infiltration and the SCNV of SMC1A. *p < 0.05; **p < 0.01; ***p < 0.001; ns—no significance.

To better understand the influences of SMC1A expression on immune cell infiltration, we performed the ssGSEA method in the TCGA database using the GSVA package to calculate the correlation coefficient between SMC1A expression and immune cells. Our results showed that high expression of SMC1A was positively correlated with immune infiltration (Figure 4A,B). We further verified the effect of SMC1A expression levels on immune cell infiltration in the mice model. Similar to the results of TCGA database, immunohistochemical data showed that in contrast to the control group, the CD45 cell infiltration was significantly increased in the SMC1A overexpression group (Figure 4C), indicating that SMC1A was positively associated with immune infiltration in the mice model. SMC1A expression showed positively associated with Th2 cells in TCGA samples (Figure 4A). Th1 and Th2 are two subtypes of CD4 T cells. Th1 cells suppress malignant tumor proliferation via inducing an immune response to tumors by mainly secreting interferon‐γ (IFN‐γ) and tumor necrosis factor‐α (TNF‐α); while Th2 cells mainly secrete interleukin‐4 (IL4), IL‐10 leading to tumor immunosuppression.^30^ Furthermore, CD4(+) regulatory T cells (Tregs) play a crucial position in immune homeostasis and contribute to tumor progression in malignancies by suppressing effective tumor immunity.^31^ Interestingly, the high expression of SMC1A was significantly linked to the expression of Tregs in TCGA database (Figure 4A). To further confirm these results, we developed the MC38 mouse model and examined the expression of Th2 and Treg cells by flow cytometry assay. Similarly, in vivo experiments also showed that the percentage of IL4^+^CD4^+^ T cells (Th2) and FoxP3^+^CD4^+^ T cells (Tregs) was significantly higher in the SMC1A overexpression group (Figure 4D,E). In conclusion, these data suggested that high SMC1A levels may be associated with tumor immunosuppression.

In addition, single‐cell database showed that SMC1A was highly expressed in proliferating T cells (Figure 3A), we therefore inquired whether SMC1A expression impacted T‐cell proliferation. Intriguingly, in vivo experiment showed that the frequency of Ki67‐positive CD3^+^ T cells was higher in SMC1A overexpressing group than in control (Figure 4F), suggesting that SMC1A expression may affect the proliferation of T cells.

In addition to gene expression, mutations in the SMC1 gene also perform key effects in the development of CRC.^14^ Next, we explored whether mutations of SMC1A were also involved in immune infiltration. The results showed that mutated SMC1A was elevated in CD8 T cells and CD8 central memory T cells (Figure 4G). Similarly, the mutated SMC1A group was significantly higher in CD4 T cells, CD4 T‐cell Th1 and Th2 than in SMC1A wild‐type group (Figure 4H). Furthermore, we also examined the correlation between immune cell infiltration and the somatic cell copy number variation (SCNV) of SMC1A. Our data revealed that B‐cell, neutrophil, and dendritic cell infiltration and the SCNV of SMC1A showed statistically significant differences; however, there was no correlation between CD8 T cells, CD4 T cells, and macrophages and CNV of SMC1A (Figure 4I).

### 3.5 SMC1A may be a predictive marker for immune checkpoint inhibitor (ICI) therapy

**FIGURE 5:**
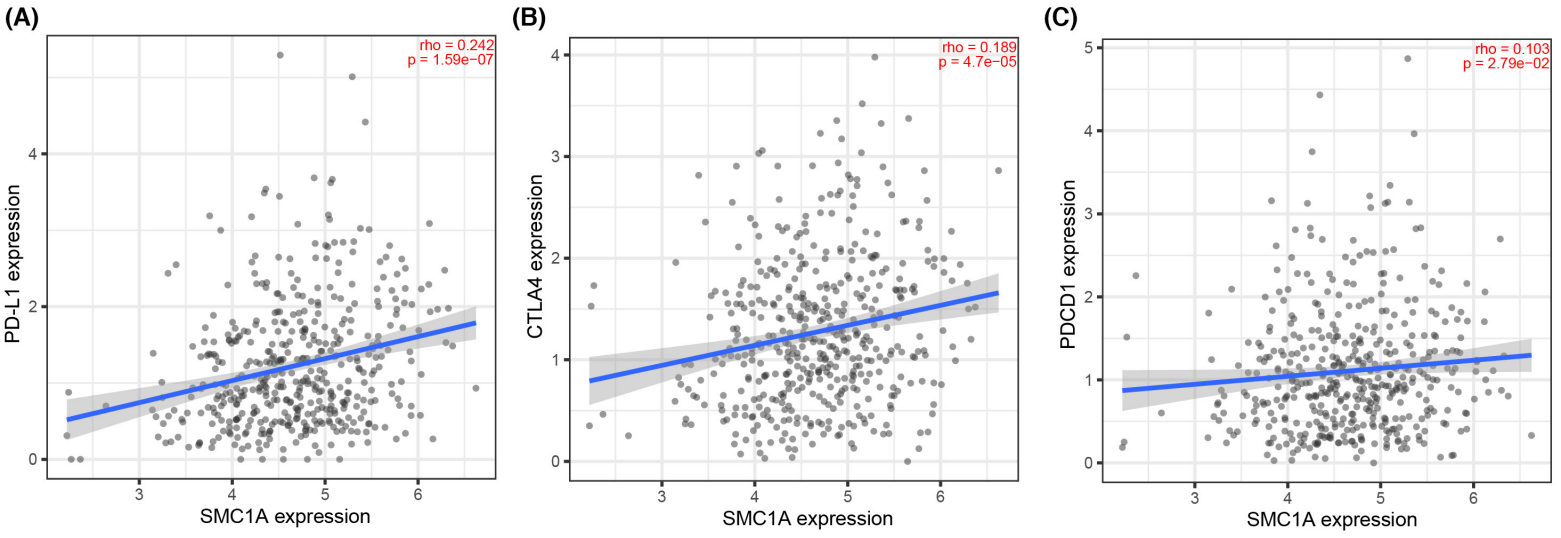
SMC1A may be a predictive marker for immune checkpoint inhibitor (ICI) therapy. The expression of SMC1A was associated with the expression of immune checkpoints PD‐L1 (A), CTLA4 (B), and PDCD1 (C) in COAD samples from TCGA database.

A large number of studies have been conducted to identify predictors of response to ICI (antiprogrammed cell death 1 (PD‐1)/programmed cell death‐ligand 1 (PD‐L1)) immunotherapy. Currently, the characteristics that predict immunotherapy response are tumor mutational burden (TMB), the expression of immune checkpoints (i.e., PD‐L1 expression), and “hot” T‐cell inflammatory microenvironment.^32^, ^33^, ^34^, ^35^ We have known that high SMC1A expression was positively correlated with immune cell infiltration, especially T‐cell immune cell infiltration, that is, SMC1A was in the “hot” T‐cell inflammatory microenvironment in colon cancer (Figure 4A,B). In addition, SMC1A was positively linked to the immune checkpoint genes CD274, CTLA4, and PDCD1 in COAD (Figure 5A–C). Therefore, we considered that SMC1A is a possible biomarker for ICI treatment prediction.

### 3.6 SMC1A may mediate tumor stem cell in COAD

**FIGURE 6:**
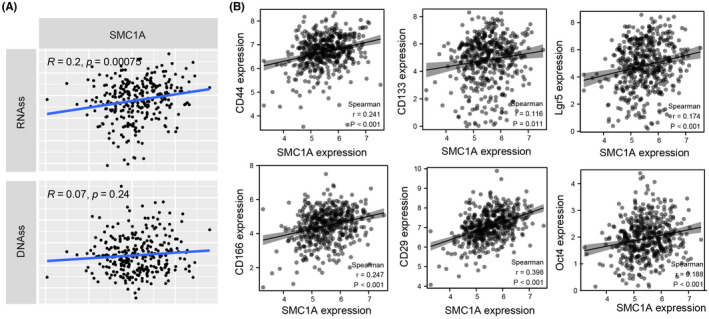
SMC1A may mediate tumor stem cells in COAD. (A) The correlation between SMC1A expression and tumor stemness scores from TCGA database. (B)The expression of SMC1A was associated with the expression of stem cell markers CD44, CD133, Lgr5, CD166, CD29, and Oct4 in COAD samples from TCGA database.

Cancer stem cells (CSCs) have been reported to show a very strong DNA damage response (DDR).^36^, ^37^ Our previous results showed that SMC1A played important roles in DNA activity (Figure 2B,C), therefore, we speculated whether SMC1A could mediate CSCs. To validate the involvement of SMC1A in the tumor stemness of COAD, we measured the mRNA levels of SMC1A with tumor stemness scores from TCGA database. We used two methods to evaluate tumor cell stemness, which were RNAss based on mRNA expression (RNA stemness score) and DNAss based on gene DNA methylation (DNA stemness score). The results showed that the expression of SMC1A and RNAss were positively correlated with COAD, which means that the higher the levels of SMC1A expression the stronger the tumor stemness (Figure 6A). However, the expression of SMC1A was not related to DNAss (Figure 6A).

Currently, the main CSC markers found to be associated with CRC are CD133, CD29, CD166, CD44, Lgr5, oct4, Nanog, etc.^38^, ^39^, ^40^, ^41^ These stem cell markers are involved in proliferation, invasive metastasis, and drug resistance of CRC via multiple molecular mechanisms.^38^, ^39^ Our association analysis indicated that the mRNA levels of SMC1A were strongly correlated with the expression levels of stem cell indicators CD133, CD29, CD166, CD44, Lgr5, and Oct4 (Figure 6B).

### 3.7 SMC1A binds to miR‐23b‐3p

**FIGURE 7:**
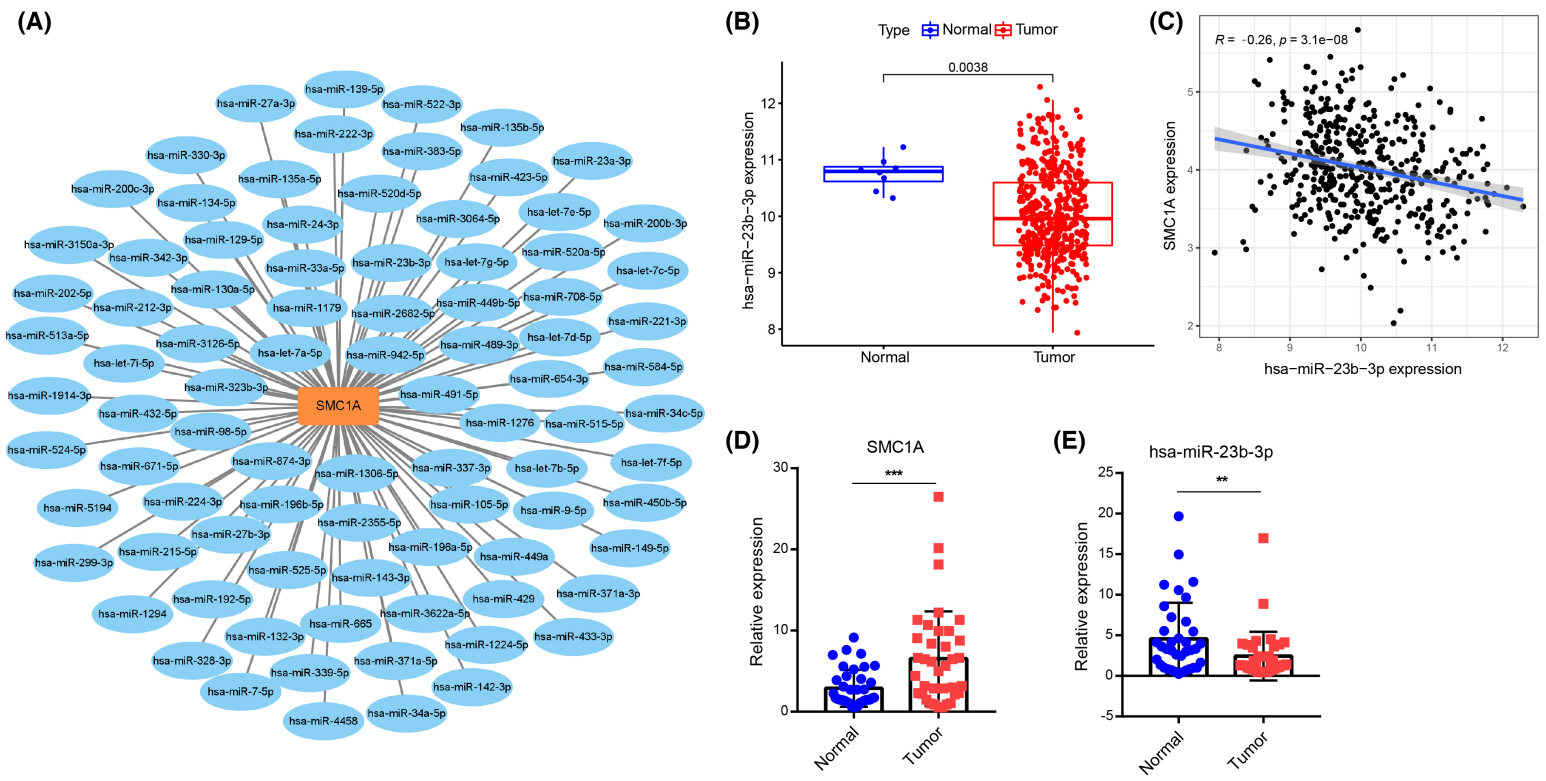
SMC1A binds to miR‐23b‐3p. (A) The binding miRNAs of SMC1A were detected in 2 and more programs from seven prediction programs (PITA, RNA22, miRmap, DIANA‐MicroT, miRanda, PicTar, and TargetScan) in ENCORI database. (B) The miR‐23b‐3p expression was significantly reduced in colon cancer tissues compared with normal tissues from TCGA database. (C) The miR‐23b‐3p and SMC1A showed a significant negative correlation of COAD samples from TCGA database. (D) The mRNA levels of SMC1A were significantly increased in colon cancer tissues compared with paraneoplastic tissues by RT‐qPCR assays in 37 clinical samples. (E) The levels of miR‐23b‐3p were significantly increased in colon cancer tissues compared with paraneoplastic tissues by RT‐qPCR assays in 37 clinical samples. **p < 0.01; ***p < 0.001.

The ceRNA mechanism of SMC1A was explored next to understand the cause of SMC1A overexpression in COAD. We investigated the binding miRNAs of SMC1A using the Encyclopedia of RNA Interactomes (ENCORI) database (https://starbase.sysu.edu.cn/). The ENCORI database contains data from CLIP‐seq, which predicts miRNA targets with Ago protein binding sites by intersections to demonstrate miRNA‐target interactions, this database provides results from seven prediction programs (PITA, RNA22, miRmap, DIANA‐MicroT, miRanda, PicTar, and TargetScan). Our results exhibited that the predicted results of binding miRNAs of SMC1A appeared in 2 and more programs (Figure 7A). We further validated the predicted miRNAs in the COAD samples from TCGA database. Among them, only miR‐23b‐3p was significantly downregulated in colon cancer tissues compared with normal tissues (Figure 7B), and miR‐23b‐3p and SMC1A showed a significant negative correlation in COAD samples (Figure 7C).

Further, we collected 37 cases of colon cancer and paired paraneoplastic tissues and performed RT‐qPCR assays for the expression levels of SMC1A and miR‐23b‐3p in the tissues. Our results showed that the mRNA levels of SMC1A were significantly increased in colon cancer tissues compared with paraneoplastic tissues (Figure 7D). However, the expression of miR‐23‐3p was significantly downregulated in 37 clinical samples (Figure 7E).

## 4 DISCUSSION

Cohesin is a complex consisting of four conserved subunits named SMC1A, SMC3, RAD21, and STAG1 or STAG2, which surround DNA within its ring structure.^42^ SMC1A is unique among the SMC family and it has been widely reported that SMC1A is involved in genomic stability maintenance and DNA repair pathways.^43^, ^44^ Recently, there is evidence that SMC1A is implicated in the pathogenesis of rare diseases and cancers.^45^ It is important to note that SMC1A functions in the DNA damage‐response pathway.^45^ Downregulation of SMC1A by oligonucleotide antisense could lead to genomic instability and chromosomal aberrations in human cells.^44^, ^46^ Moreover, SMC1A is phosphorylated at Ser957 and Ser966 residues after DNA damage resulting from chemical treatment or ionizing radiation.^45^ In this study, we also showed that functional and pathway enrichment analysis of SMC1A showed a strong correlation with DNA activity, which was consistent with previous reports.

Since proliferating cells must rapidly repair DNA damage, this is even more important for T cells from which invading pathogens must be rapidly removed.^47^, ^48^ However, there is a very short cell cycle in activated T cells, which makes them extremely susceptible to DNA damage.^49^ For this reason, T cells must respond robustly and rapidly to DDR to repair DNA damage.^50^ Although there is evidence that DDR can be induced by the activation of T cells, little is known about how the DDR is regulated in proliferating T cells. Our previously published results demonstrated that interference with SMC1 expression was able to increase the sensitivity of colon cancer cells to oxaliplatin, which was attributed to the regulation of SMC1 on DDR.^16^ In the present study, we found that SMC1A was expressed in a multitude of immune cells, especially in proliferating T cells, and SMC1A expression in colon cancer could affect T‐cell proliferation. We therefore speculate that SMC1A may be an important modulator of T cells that may regulate proliferation in response to DNA damage. Asif et al. reported that SMC1A interacts with E3 ubiquitin ligase Cullin‐4b (Cul4b) allowing the survival and proliferation of activated T cells to promote the repair of DNA damage,^49^ which supports our speculation.

With the development of research in recent years, there have been advances in both diagnosis and treatment of CRC. Immunotherapy has become a new direction in the treatment of CRC, and relevant clinical trials of immunotherapy are being conducted.^51^, ^52^ However, it is to be noted that CRC is one of the tumors with poor immunotherapeutic results.^53^ Hence, it is particularly important to find predictive markers of the effectiveness of ICIs and to find the appropriate population for ICIs in CRC patients. To determine the effectiveness of ICIs, there is currently a huge amount of studies achieving consensus on several aspects, which are TMB, expression of immune checkpoints (e.g., PD‐L1 expression), and the “hot” T‐cell inflammatory microenvironment.^32^, ^33^, ^34^, ^35^ High TMB, greater PD‐L1 expression, and higher T‐cell infiltration benefit from ICI therapy.^32^, ^34^, ^35^ Here, the correlation between SMC1A and immune cell infiltration was further investigated, and high expression of SMC1A showed a high immune cell infiltration status. This implies that SMC1A presented a positive correlation with the “hot” immune environment in colon cancer. The SMC1A levels were directly positive associations with the immune checkpoint genes CD274, CTLA4, and PDCD1 in COAD patients. Therefore, we proposed that SMC1A may become a biomarker for the treatment prediction of ICIs.

CSCs are a subgroup of tumors that are ascribed to tumor metastasis and resistance to chemotherapy and radiotherapy, and eventually result in tumor recurrence.^54^ Kristiaan J Lenos et al. reported that colon cancer relies on CSCs for expansion and recurrence after treatment, and that these stem cells driving colon cancer are mainly located at the tumor margin, in proximity to tumor‐associated fibroblasts.^55^ CSCs have been found to act as protectors of autophagy and potent cellular recycling, as well as highly competent regulators of epithelial‐mesenchymal transition (EMT), scavengers of reactive oxygen species (ROS), and DNA repair systems.^56^ SMC1A as a factor in regulating DDR, we also found that SMC1A plays a positive correlation with the induction of CSCs. A few reports showed that SMC1 is crucial in coordinating the local OCT4 chromatin structure, thereby inducing pluripotent stemness of cells,^57^, ^58^ which are consistent with our findings.

Recently, noncoding RNA (ncRNA) has been shown to be associated with the occurrence and progression of colon cancer.^59^ As is known, ncRNAs are part of a subclass of transcripts that are primarily translated into proteins, but they also exert critical functions in various cytosolic and physiological processes.^60^ MicroRNAs (miRNAs) are a class of ncRNA molecules of approximately 22 nucleotides in length. miRNAs usually act as competing endogenous RNAs (ceRNAs) to regulate the expression of specific genes.^61^, ^62^ Our results showed that miR‐23b‐3p binds SMC1A, but we still need more cellular experiments for further validation.

Our previous results suggested that SMC1A could potentially serve important roles in proliferation and drug resistance in CRC, and SMC1A could be applied as a poor prognostic indicator for CRC patients. However, it was rarely reported that SMC1A also acted as essential effects on tumor immune infiltration and CSCs in CRC. The present study expanded new investigation approaches, which will provide more theoretical basis for the clinical application of SMC1A in CRC. However, this topic still has some limitations. Firstly, whether and how SMC1A is capable of regulating DDR in proliferating T cells. Moreover, whether SMC1A acts as a predictive biomarker for the treatment of ICIs in CRC. We need further validation with mouse experiments. Finally, more clinical tissue specimens and in vitro and in vivo experiments are needed to validate the correlation between SMC1A and CSCs in CRC.

In conclusion, SMC1A may be a bidirectional target switch that simultaneously regulates the immune microenvironment and tumor stem cells. Moreover, SMC1A may be a biomarker for the prediction of ICI therapy.

## AUTHOR CONTRIBUTIONS

**Jin Li:** Data curation (lead); formal analysis (lead); funding acquisition (lead); investigation (lead); methodology (lead); writing – original draft (lead). **Qian Zhou:** Data curation (supporting); investigation (supporting); methodology (supporting); writing – review and editing (supporting). **Li Liu:** Data curation (supporting); writing – review and editing (supporting). **Jing‐dong He:** Data curation (supporting); supervision (lead); validation (lead); writing – review and editing (lead).

## FUNDING INFORMATION

This study was supported by the project fund of Nanjing Medical University (NMUB2018149) and the project fund of the Science and Technology Bureau of Huai'an (No. HAB202018).

## CONFLICT OF INTEREST STATEMENT

These authors have no conflict of interest to declare.

## CLINICAL TRIAL REGISTRATION

All procedures were approved by the committee of The Affiliated Huaian No.1 People's Hospital of Nanjing Medical University. The clinical trial registration number is YX‐2021‐106‐01.

## ETHICS APPROVAL STATEMENT

We declare that the study was conducted in a manner that posed no harm or risk to the subjects and recruitment of subjects was completely voluntary and informed. All procedures involving human material in this study were approved by the committee of The Affiliated Huaian No.1 People's Hospital of Nanjing Medical University.

## PATIENT CONSENT STATEMENT

All patients signed the informed consent forms. All samples used in this project were de‐identified and assigned a study number.

## Data Availability

The datasets used and/or analyzed during the current study are available from the corresponding authors upon reasonable request.
